# What do Cochrane systematic reviews say about interventions for age-related macular degeneration?

**DOI:** 10.1590/1516-3180.2019.010317092019

**Published:** 2020-03-06

**Authors:** Vania Mozetic, Rafael Leite Pacheco, Carolina de Oliveira Cruz Latorraca, Fernanda Chin Yu Ogasawara Lee, João Victor Borges Gomes, Rachel Riera

**Affiliations:** I MD. Ophthalmologist, Instituto Dante Pazzanese de Cardiologia, São Paulo (SP), Brazil.; II MD. Researcher, Centro Universitário São Camilo, and Master’s Student, Evidence-Based Health Program, Universidade Federal de São Paulo (UNIFESP), São Paulo (SP), Brazil.; III MSc. Psychologist and Doctoral Student, Evidence-Based Health Program, Universidade Federal de São Paulo (UNIFESP), and Assistant Researcher, Cochrane Brazil, São Paulo (SP), Brazil.; IV Undergraduate Medical Student, Escola Paulista de Medicina (EPM), Universidade Federal de São Paulo (UNIFESP), São Paulo (SP), Brazil.; V Undergraduate Medical Student, Escola Paulista de Medicina (EPM), Universidade Federal de São Paulo (UNIFESP), São Paulo (SP), Brazil.; VI MD, MSc, PhD. Rheumatologist and Adjunct Professor, Discipline of Evidence-Based Medicine, Escola Paulista de Medicina (EPM), Universidade Federal de São Paulo (UNIFESP), and Coordinator, Centre of Health Technology Assessment, Hospital Sírio-Libanês, São Paulo (SP), Brazil.

**Keywords:** Review [publication type], Macular degeneration, Macular degeneration, age-related, 1 [supplementary concept], Evidence-based medicine, Evidence-based practice, Cochrane reviews, Age-related macular degeneration, Systematic reviews, Overview

## Abstract

**BACKGROUND:**

Age-related macular degeneration (AMD) is the third largest cause of blindness worldwide, accounting for 8.7% of all cases. A considerable number of preventive or therapeutic interventions have been used for AMD.

**OBJECTIVE:**

This study presents a critical view of the interventions that have been assessed through Cochrane systematic reviews.

**DESIGN AND SETTING:**

Review of systematic reviews, conducted in the Discipline of Evidence-Based Medicine, Escola Paulista de Medicina (EPM), Universidade Federal de São Paulo (UNIFESP).

**METHODS:**

Review of Cochrane systematic reviews about interventions for AMD.

**RESULTS:**

The 18 systematic reviews included assessed the effects of surgical techniques, laser/photo/radiotherapy, intravitreal injections, systemic drugs and phytotherapy/vitamins/supplements.

**CONCLUSION:**

The Cochrane systematic reviews found evidence that use of bevacizumab, ranibizumab, pegaptanib, laser photocoagulation, photodynamic therapy and multivitamin compounds may present some benefits for treating AMD. There was insufficient evidence for supporting the use of macular translocation, submacular surgery, steroid implantation, radiotherapy, intravitreal aflibercept, interferon alfa, statins or omega-3 fatty acids for treating AMD; or the use of multivitamin antioxidant vitamins or mineral supplementation for preventing AMD. Future randomized controlled trials are imperative to reduce the uncertainty in several clinical questions regarding AMD.

## INTRODUCTION

Age-related macular degeneration (AMD) is a degenerative disease of the macula (central region of the retina) that causes loss of central vision. This type of vision is essential for performing activities of daily living.^1^

AMD is the third largest cause of blindness worldwide, accounting for 8.7% of all cases of definitive loss of vision.^1^ Currently, 15% to 24% of the population over the age of 65 years are affected by the early stages of AMD.^2^

AMD is differentiated into the early (often asymptomatic) or intermediate stages with drusen (amorphous extracellular sediments in the retina) and characteristic pigmentary changes, and the late stages. For clinical purposes, the late stages of AMD have been classified as dry (non-neovascular and atrophic) or wet (neovascular and exudative). In the wet stages, new blood vessels can lead to leakage and tissue lesions.^3^ Although the neovascular form represents only 10% of the disease burden, it is responsible for 90% of AMD-related blindness.

A considerable number of preventive or therapeutic interventions are available and have been used for both types of AMD. This study presents a critical view of the interventions that have been assessed through Cochrane systematic reviews (SRs).

## OBJECTIVE

To synthetize and present the results from Cochrane SRs assessing interventions for preventing and treating age-related macular degeneration.

## METHODS

### Design and setting

We carried out a narrative review of Cochrane SRs in the Discipline of Evidence-Based Medicine of Escola Paulista de Medicina (EPM), Universidade Federal de São Paulo (UNIFESP). This manuscript was elaborated for the section Cochrane Highlights. This initiative is a formal collaboration between the São Paulo Medical Journal and Cochrane, and it is supported by Cochrane Brazil. The aim of this initiative is to disseminate the evidence from Cochrane SRs.

### Inclusion criteria

#### Types of studies

We included only the latest published version of Cochrane SRs. We did not consider protocols, or any SR marked as “withdrawn” in the Cochrane Database of Systematic Reviews (CDSR).

#### Types of participants

In relation to reviews examining therapeutic methods, we considered any participant with the diagnosis of AMD, as defined by the review authors’ criteria. SRs including cases of AMD and other clinical situations were included only if the subset of data on AMD participants was provided separately. In relation to reviews examining preventive methods, no restrictions on participants were applied.

#### Types of intervention

We considered any surgical or pharmacological (local or systemic) intervention, compared with placebo, no intervention or any other intervention.

#### Type of outcomes

We considered all clinical and laboratory outcomes addressed by the SRs.

### Search for reviews

We carried out a systematic search in the Cochrane Database of SRs (via Wiley) on January 8, 2019. The search strategy is presented in Table 1.

**Table 1.** Search strategy

#1 MeSH descriptor: [Macular Degeneration] explode all trees
#2 (Maculopathies, Age-Related) or (Macular Degeneration, Age-Related) or (Age Related Maculopathies) or (Age Related Maculopathy) or (Macular Degenerations, Age-Related) or (Age-Related Macular Degeneration) or (Macular Dystrophies) or (Dystrophies, Macular) or (Degeneration, Macular) or (Age-Related Macular Degenerations) or (Age-Related Maculopathy) or (Macular Degenerations) or (Maculopathies, Age Related) or (Dystrophy, Macular) or (Age-Related Maculopathies) or (Maculopathy, Age-Related) or (Macular Dystrophy) or (Degenerations, Macular) or (Degenerations, Age-Related Macular) or (Age Related Macular Degeneration) or (Degeneration, Age-Related Macular) or (Maculopathy, Age Related)
#3 #1 or #2
Filters: in Cochrane Reviews; *in Title, Abstract, Keywords*

### Selection of systematic reviews

The selection process was performed by two authors (RLP and RR), who independently assessed all titles and abstracts that had initially been obtained through the electronic search for potential reviews. These authors confirmed the eligibility of these SRs by assessing their full texts. Any divergences in the selection process were resolved through reaching a consensus.

### Presentation of the results

We summarized and presented the following characteristics from the SRs that were included: PICOs (population, intervention, comparator and outcomes), goals, methods, main findings, certainty of evidence in accordance with the GRADE approach (Grading of Recommendations Assessment, Development and Evaluation)^4^ and conclusions.

## RESULTS

### Search results

The initial search retrieved 90 abstracts of systematic reviews (SRs), and 18 of them fulfilled our inclusion criteria and were considered for the analysis.^5^^,^^6^^,^^7^^,^^8^^,^^9^^,^^10^^,^^11^^,^^12^^,^^13^^,^^14^^,^^15^^,^^16^^,^^17^^,^^18^^,^^19^^,^^20^^,^^21^^,^^22^

### Results from systematic reviews

The 18 SRs included assessed the effects of surgical techniques (n = 4),^5^^,^^6^^,^^7^^,^^8^ laser/photo/radiotherapy (n = 4),^9^^,^^10^^,^^11^^,^^12^ intravitreal injections (n = 3),^13^^,^^14^^,^^15^ systemic drugs (n = 3)^16^^,^^17^^,^^18^ and phytotherapy/vitamins/supplements (n = 4)^19^^,^^20^^,^^21^^,^^22^ for preventing AMD or treating participants with AMD. The main results from the SRs that were included and the certainty of the evidence (based on the GRADE approach)^4^ are presented in Table 2. A brief narrative synthesis of each SR is presented below.

**Table 2.** Characteristics, main results and certainty of evidence of the systematic reviews included

Surgical interventions				
Intervention	Population (sample)	Comparison	Main findings	Certainty of evidence (GRADE)*
Full macular translocation^6^	AMD (n = 50)	Full macular translocation versus PDT	Benefit of macular translocation: • Gain of three or more lines in the ETDRS test • Change of visual acuity • Change of near visual acuity score No difference between interventions groups: • Progression to loss of vision • Change of contrast sensitivity • Recurrence of choroidal neovascularization Benefit of PDT: • Complications were minor and less frequent in PDT Time point for measurement: 12 months	NA
Submacular surgery^7^	AMD with or without blood in the macula (n = 890)	Submacular surgery versus observation	No difference between groups: • Progression to loss of vision • Visual gain Benefit of observation: • Cataracts needing surgery • Retinal detachment Time point for measurement: 12 months	High Low NA NA
Steroid implantation (intra‐ and peri‐ocular)^8^	AMD (n = 809)	Anecortave acetate versus placebo	• Progression to loss of vision: benefit over placebo for anecortave 15 mg**, but not for 3 mg or for 30 mg	NA
		Triamcinolone acetonide versus placebo	• Progression to loss of vision: no difference between groups	NA
		Anecortave acetate versus PDT	• Progression to loss of vision: no difference between groups Time point for measurement: 12 months	NA
Laser/photo/radiotherapy				
Intervention	Participants	Comparisons	Main findings	Certainty of evidence (GRADE)*
Laser photocoagulation	AMD with drusen (n = 2,159/3,580 eyes)^9^	Laser versus no intervention	Benefit of laser: • Reduction of drusen • Risk of choroidal neovascularization • Risk of geographic atrophy • Progression to loss of vision	High High Low Moderate
	AMD (n = 2,064)^10^	Direct photocoagulation of the entire choroidal neovascularization versus no intervention	Benefit of photocoagulation: • Progression to loss of vision at 24 months Benefit of no intervention: • Progression to loss of vision at three months	NA
		Perifoveal photocoagulation versus observation	Benefit of photocoagulation: • Progression to loss of vision at 24 months	NA
PDT^11^	AMD (n = 1,429)	PDT with verteporfin versus PDT with 5% dextrose in water	Benefit of PDT/verteporfin • Progression to loss of vision Time point for measurement: 12 and 24 months No difference between groups: • Risk of severe decrease in visual acuity Time point for measurement: one week Benefit of PDT/dextrose: Infusion-related back pain: higher with PDT/verteporfin (RR 9.93; 95% CI 2.82 to 35.02; 4 RCTs; 1439 participants; high certainty of evidence) Time point for measurement: one week	High Moderate High
Radiotherapy^12^	AMD (n = 1,154)	External beam radiotherapy or plaque brachytherapy versus no intervention	Benefit of radiotherapy: • Progression of loss of vision at 24 months (only considering loss of six or more lines) No difference between groups: • Progression of loss of vision at 24 months	Low Moderate
Intravitreal injections of anti-VEGF				
Intervention	Participants	Comparisons	Main findings	Certainty of evidence (GRADE)*
Aflibercept^13^	Neovascular AMD with active subfoveal choroidal neovascular lesions (n = 2,412)	Aflibercept versus no intervention	No difference between groups: • Change in best‐corrected visual acuity (BCVA) • Gain of 15 or more letters of BCVA • Loss of 15 or more letters of BCVA • Serious systemic adverse events Time points for measurement: 12 and 24 months	NA High High Moderate
Bevacizumab ***^14^	AMD (n = 159)	Bevacizumab versus standard therapy	Benefit of bevacizumab: • Gain of 15 or more letters of visual acuity at one year • Progression to loss of vision No difference between groups: • Serious systemic adverse events	Moderate Moderate Low
Ranibizumab***^14^	AMD (n = 1,322)	Ranibizumab versus sham	Benefit of ranibizumab: • Progression to loss of vision at one year • Serious adverse events	High Moderate
Pegaptanib^14^	AMD (n = 1,186)	Pegaptanib versus sham	Benefit of pegaptanib: • Gain of 15 or more letters of visual acuity at one year • Loss of fewer than 15 letters of visual acuity at one year No difference between groups: • Serious adverse events	High High Moderate
Systemic medication				
Intervention	Population	Comparisons	Main findings	Certainty of evidence (GRADE)*
Interferon alpha^17^	AMD (481 participants)	Interferon alpha versus placebo	Benefit of placebo: • Progression to loss of vision at 52 weeks	NA
Statins^18^	Older people at high risk of developing AMD (drusen observed) (n = 144)	Simvastatin versus placebo	No difference between groups: • Visual acuity at three months of treatment, and at 45 days and 12 months after the completion of treatment • Drusen score at 12 months • Progression of AMD at 36 months • Adverse events	NA NA Low NA
Phytotherapy/vitamins/supplements				
Intervention	Population	Comparisons	Main findings	Certainty of evidence (GRADE)*
Beta-carotene	Healthy individuals (n = 22,083)^19^	Beta-carotene versus placebo	No difference between groups: • Overall risk of AMD • Risk of late AMD Harm with beta-carotene: • Risk of lung cancer in people who smoked	High Moderate High
Lutein and/or zeaxanthin	AMD^20^	Lutein and/or zeaxanthin versus placebo	No difference between groups: • Progression to late AMD • Progression to loss of vision • Quality of life evidence • Mortality	Low Low Moderate Very low
Multivitamins	Healthy men (n = 14,233)^19^		Harm from multivitamins: • Overall risk of AMD • Risk of skin rashes No difference between groups: • Risk of late AMD	Moderate Moderate Moderate
	AMD (n = 2,445)^20^		Benefit of multivitamins: • Progression to late AMD • Progression to loss of vision • Quality of life No difference between groups: • Mortality Harm from multivitamins: • Risk of yellow skin	Moderate Moderate Very low Very low
Omega-3 fatty acids^22^	AMD (n = 2,343)		No difference between groups: • Progression to advanced AMD • Progression to loss of vision at 24 and 36 months • Adverse events	High Moderate High
Vitamin C	Healthy men (n = 14,236)^19^	Vitamin C versus placebo	No difference between groups: • Overall risk of AMD • Risk of late AMD	High Moderate
Vitamin E	Healthy individuals (n = 55,614)^19^	Vitamin E versus placebo	No difference between groups: • Overall risk of AMD: • Risk of late AMD • Overall risk of adverse events Harm from vitamin E: • Risk of hemorrhagic stroke	High Moderate NA Low
	AMD (n = 998)^20^	Vitamin E versus placebo	No difference between groups: • Progression to late AMD • Progression to loss of vision • Withdrawal due to adverse events No serious adverse events	Very low Low Very low
Zinc^20^	AMD (n = 3,790)	Zinc versus placebo	Benefit from zinc: • Progression to late AMD • Progression to loss of vision	Low Moderate

AMD = age-related macular degeneration; ETDRS = Early Treatment Diabetic Retinopathy Study; NA = not assessed; PDT = photodynamic therapy; RCTs = randomized clinical trials; VEGF = anti-vascular endothelial growth factor.

*GRADE (Grading of Recommendations Assessment, Development and Evaluation). This system assesses the certainty of the body of evidence. High certainty of evidence means that there is high confidence that the estimated effect is near to the true effect; moderate certainty means that it is very likely that the estimated effect is close to the real effect, but there is a possibility that it is not; low certainty means that there is only limited confidence in the effect estimate; and very low certainty means that the true effect is likely to be substantially different from the estimated effect.

**statistical benefit (clinical benefit is questionable); ***for ranibizumab versus bevacizumab, see the reviews relating to this, in the text.

### Surgical techniques

#### 1. Implantable mini-telescope for diminishing loss of vision

A mini-telescope is an implantable ophthalmic device for amending visual acuity after impairment of vision due to AMD. It has been used to enlarge objects in the central visual field and focus them onto healthy areas of the retina, thus making it possible to view objects that otherwise could not be seen. This review^5^ assessed the effects of an implantable ophthalmic mini-telescope in individuals with late or advanced AMD, but no randomized clinical trials (RCTs) or quasi‐RCTs were found.

The authors could not draw any conclusions regarding this clinical question. There is one ongoing RCT that is comparing the OriLens intraocular telescope with standard low-vision training for coping with end‐stage AMD. The results from this trial are expected in 2020.

For further details and to access all the analyses, see the original abstract, available from: https://www.cochranelibrary.com/cdsr/doi/10.1002/14651858.CD011140.pub2/full.

#### 2. Macular translocation

Macular translocation is a surgical procedure that includes displacement of the retina to a less‐damaged area, which could improve vision. This review^6^ aimed to assess the effects of this procedure for maintaining or improving vision in patients with AMD. The authors found only one small open study (n = 50) that compared full macular translocation versus photodynamic therapy (PDT) for AMD. After one year, macular translocation presented some benefit regarding the following outcomes:

- Gain of three or more lines read during the ETDRS (Early Treatment Diabetic Retinopathy Study) test (risk ratio [RR] 21; 95% confidence interval [CI] 1.30 to 340.02);
- Change of visual acuity (mean difference [MD] 14.60; 95% CI 5.39 to 23.81);
- Change of near visual acuity score (MD 17.80; 95% CI 3.98 to 31.62).

However, there was no difference between the interventions groups regarding these other outcomes:

- Progression of loss of vision (loss of three or more lines) (RR 0.56; 95% CI 0.22 to 1.43);
- Change of contrast sensitivity (MD: one letter favoring translocation; 95% CI ‐3.51 to 5.51);
- Recurrence of choroidal neovascularization (RR 1.56; 95% CI 0.83 to 2.91).

Complications were minor and less frequent in the PDT group. The complications observed in the macular translocation group included: retinal detachment (6/25 patients), diplopia requiring prismatic correction (5/25 patients), macular edema (11 eyes; six of them required surgery for retinal detachment) and need for muscle surgery (23 eyes).

The authors concluded that the current evidence was insufficient for them to be able to recommend macular translocation for AMD, which is also associated with significant harm. This technique is complicated and long surgical training is needed in order to be able to perform it.

For further details and to access all the analyses, see the original abstract, available from: https://www.cochranelibrary.com/cdsr/doi/10.1002/14651858.CD006928.pub2/full.

#### 3. Submacular surgery for choroidal neovascularization

Surgical removal of the submacular tissue underlying the macula (within which small new blood vessels grow) might limit the development of AMD. This review^7^ aimed to assess the effectiveness of submacular surgery for preserving or improving vision in individuals with AMD and included three RCTs. Two RCTs comparing submacular surgery for AMD with observation were found, including patients with (n = 336) or without (n = 454) blood in the macula. After one year, no difference was found between the intervention arms in relation to:

- Prevention of loss of vision (RR: 0.96; 95% CI 0.84 to 1.09; risk difference [RD] ‐2%; 95% CI ‐10% to 5%; excluding a large benefit from surgery, in terms of absolute risk in this sample; high certainty of evidence);
- Probability of visual gain (RR: 1.06; 95% CI 0.75 to 1.51; RD 1%; 95% CI ‐4% to 6%; excluding a large benefit from surgery, in terms of absolute risk in this sample; low certainty of evidence).

However, cases of cataracts requiring surgery (RR: 8.69; 95% CI: 4.06 to 18.61) and retinal detachment (RR: 6.13; 95% CI: 2.81 to 13.38) were more frequent in the surgical group. Detachment was observed in 5% of the participants without extensive blood under the macula and in 18% of those with this.

In another small pilot RCT, submacular surgery was compared with laser photocoagulation (n = 70) and no difference was found for any of the outcomes measured.

The authors of the SR concluded that submacular surgery for choroidal neovascularization did not provide any benefit for individuals with AMD.

For further details and to access all the analyses, see the original abstract, available from: https://www.cochranelibrary.com/cdsr/doi/10.1002/14651858.CD006931.pub2/full.

#### 4. Steroid implantation (intra‐ and peri‐ocular)

Steroids have anti‐inflammatory and antiangiogenic properties that can be useful for treating AMD. This review^8^ aimed to assess the effects of intra‐ and peri‐ocular antiangiogenic steroids for treating neovascular AMD. Three clinically heterogeneous RCTs (809 participants) were found, comparing: (a) different doses of anecortave acetate versus placebo; (b) triamcinolone acetonide versus placebo; and (c) anecortave acetate versus photodynamic therapy (PDT). For the main outcome, i.e. progression to loss of vision (loss of three or more lines of vision), the results at 12 months were the following:

- Anecortave acetate (3 mg) versus placebo: no difference between the interventions (RR 0.8; 95% CI 0.45 to 1.45);
- Anecortave acetate (15 mg) versus placebo: slight difference favoring steroids, but clinical relevance needs to be discussed (RR 0.45; 95% CI 0.21 to 0.97);
- Anecortave acetate (30 mg) versus placebo: no difference between the interventions (RR 0.91; 95% CI 0.52 to 1.58);
- Triamcinolone acetonide versus placebo: no difference between the interventions (RR 0.97; 95% CI 0.74 to 1.26);
- Anecortave acetate versus PDT: no difference between the interventions (RR 1.08; 95% CI 0.91 to 1.29)

The authors of the SR did not find any evidence that antiangiogenic steroids prevented loss of vision due to AMD.

For further details and to access all the analyses, see the original abstract, available from: https://www.cochranelibrary.com/cdsr/doi/10.1002/14651858.CD005022.pub3/full.

### Laser/photo/radiotherapy

#### 5. Laser photocoagulation of drusen in AMD

Drusen, which consist of an amorphous yellowish aggregate that accumulates under the retina, are considered to be risk factors for developing AMD. This review^9^ evaluated the effects of laser photocoagulation of drusen in AMD and found 11 RCTs (2159 participants; 3580 eyes) comparing laser with control (no intervention). Overall, the risk of bias in the studies included was low. The secondary outcome of “probability of reducing the drusen” was reached more frequently with laser (OR 9.16; 95% CI 6.28 to 13.4; 3 RCTs; 570 participants; 944 eyes; high certainty of evidence). The results relating to primary and other secondary outcomes showed that there was no benefit from laser, considering the following:

- Risk of choroidal neovascularization at two years of follow-up: odds ratio (OR) 1.07; 95% CI 0.79 to 1.46; eleven RCTs; 2159 participants; 3580 eyes; high certainty of evidence.
- Risk of geographic atrophy: OR 1.30; 95% CI 0.38 to 4.51; two RCTs; 148 participants; 148 eyes; low certainty of evidence.
- Progression to loss of vision (loss of three or more lines of visual acuity): OR 0.99; 95% CI 0.81 to 1.22; nine RCTs; 2002 participants; 2386 eyes; moderate certainty of evidence.

No further adverse events (apart from development of choroidal neovascularization, geographic atrophy or loss of vision) were reported.

The authors of this SR concluded that laser photocoagulation of drusen led to their disappearance but did not reduce the risk of developing choroidal neovascularization, geographic atrophy or loss of visual acuity. Ongoing RCTs are being conducted to evaluate the effects of extremely short laser pulses (i.e. nanosecond laser treatment) and the results will be available in the future.

For further details and to access all the analyses, see the original abstract, available from: https://www.cochranelibrary.com/cdsr/doi/10.1002/14651858.CD006537.pub3/full.

#### 6. Laser photocoagulation for AMD

This review^10^ aimed to assess the effects of laser photocoagulation for treating neovascular AMD and included 15 RCTs (2,064 participants) assessing the following: direct photocoagulation of the entire choroidal neovascularization (11 RCTs); perifoveal photocoagulation (one RCT); and grid photocoagulation (three RCTs). In 12 trials, the control group consisted of observation alone.

In comparing direct photocoagulation of the entire choroidal neovascularization versus no intervention, the risk of progression to loss of vision (loss of six or more lines of visual acuity) was found to be more frequent in the photocoagulation group at three months (RR 1.41; 95% CI 1.08 to 1.82), but less frequent at two years (RR 0.67; 95% CI 0.53 to 0.83). In comparing perifoveal photocoagulation versus observation, a benefit from the intervention was observed at two years (RR 0.36; 95% CI 0.18 to 0.72). For other comparisons, no other differences were found.

For further details and to access all the analyses, see the original abstract, available from: https://www.cochranelibrary.com/cdsr/doi/10.1002/14651858.CD004763.pub2/full.

#### 7. Photodynamic therapy (PDT)

PDT has been investigated as an option for managing neovascular membranes in cases of AMD without affecting the retina. This review^11^ aimed to assess the effects of PDT for neovascular revascularization in patients with AMD and included four trials (1429 participants) comparing PDT with verteporfin versus PDT with 5% dextrose in water. Verteporfin (Visudyne) is a benzoporphyrin derivative that is used as a photosensitizer during PDT. The main findings from this review were the following:

- Progression of loss of vision at 24 months:
  - Loss of three or more lines of visual acuity: benefit with PDT/verteporfin (RR 0.80; 95% CI 0.73 to 0.88; four RCTs; 1381 participants; high certainty of evidence);
  - Loss of six or more lines on visual acuity test: benefit with PDT/verteporfin (RR 0.66; 95% CI 0.56 to 0.83; four RCTs; 1381 participants; high certainty of evidence).

The results at 12 months were similar to those at 24 months.

- Adverse outcome, within one week of treatment:
  - Risk of severe decrease of visual acuity: no difference between interventions (RR 3.75; 95% CI 0.87 to 16.12; three RCTs; 1075 participants; moderate certainty of evidence);
  - Infusion-related back pain: higher with PDT/verteporfin (RR 9.93; 95% CI 2.82 to 35.02; four RCTs; 1439 participants; high certainty of evidence).

Two other trials compared different treatment regimens: (a) standard versus delayed light application; and (b) retreatment every two months versus every three months. No difference in effectiveness was found in either of these.

For further details and to access all the analyses, see the original abstract, available from: https://www.cochranelibrary.com/cdsr/doi/10.1002/14651858.CD002030.pub3/full.

#### 8. Radiotherapy

This review^12^ had the aim of assessing the effects of radiotherapy for treating AMD. The review included 13 RCTs (1,154 participants) on external beam radiotherapy (dosages from 7.5 to 24 Gy) and one RCT (n = 88) on plaque brachytherapy (15 Gy at 1.75 mm for 54 minutes/12.6 Gy at 4 mm for 11 minutes). The main findings comparing radiotherapy versus control were:

- Progression of loss of vision:
  - Loss of three or more lines on visual acuity test: no difference between the groups at 24 months (RR 0.8; 95% CI 0.63 to 1.03); four RCTs, 428 participants; low certainty of evidence) or at 12 months (RR 0.90; 95% CI 0.74 to 1.1; eight RCTs; 759 participants; moderate certainty of evidence);
  - Loss of six or more lines on visual acuity test: no difference between the groups at 24 months (RR 0.81; 95% CI 0.64 to 1.03; four RCTs; 428 participants; moderate certainty of evidence), but lower occurrence with radiotherapy at 12 months (RR 0.81; 95% CI 0.44 to 0.87; seven RCTs; 576 participants; low certainty of evidence).

The frequency of adverse events was low and there were no reports of radiation retinopathy, optic neuropathy or malignancy.

The authors of this SR concluded that there was no convincing evidence that radiotherapy was an effective treatment for neovascular AMD.

For further details and to access all the analyses, see the original abstract, available from: https://www.cochranelibrary.com/cdsr/doi/10.1002/14651858.CD004004.pub3/full.

### Intravitreal injections

#### 9. Aflibercept

Aflibercept is a biological drug that blocks the biological activity of vascular endothelial growth factor (VEGF) and inhibits abnormal growth of blood vessels. This review^13^ assessed the effects of use of aflibercept for treating patients with AMD. Two RCTs, supported by the company that manufactures aflibercept, and comprising 2,457 participants with active subfoveal choroidal neovascular lesions, were included. The main findings were the following:

- Change in best‐corrected visual acuity (BCVA): no difference between the groups at one year (MD ‐0.15 in Early Treatment Diabetic Retinopathy Study [ETDRS] letters; 95% CI -1.47 to 1.17; two RCTs; 2412 participants; high certainty of evidence); and insufficient results for assessing the outcome at two years (MD 0.7 in ETDRS letters, but the data available were insufficient for calculation of the CI).
- Gain of 15 or more letters in BCVA test: no difference between the groups at one year (RR 0.97; 95% CI 0.85 to 1.11; two RCTs; 2412 participants; high certainty of evidence) or at two years (RR 0.98; 95% CI 0.85 to 1.12; two RCTs; 2412 participants; high certainty of evidence).
- Loss of 15 or more letters in BCVA test: no difference between the groups at one year (RR 0.89, 95% CI 0.61 to 1.30; two RCTs, 2412 participants; high certainty evidence).
- Serious systemic adverse events: no difference between the groups at one year (RR 0.99; 95% CI 0.79 to 1.25; two RCTs; 2419 participants; moderate certainty of evidence).
- Any serious ocular adverse event: no difference between the groups (RR 0.62; 95% CI 0.36 to 1.07; two RCTs; 2419 participants; moderate certainty of evidence).

For further details and to access all the analyses, see the original abstract, available from: https://www.cochranelibrary.com/cdsr/doi/10.1002/14651858.CD011346.pub2/full.

#### 10. Anti‐vascular endothelial growth factor

This review^14^ assessed the use of anti-vascular endothelial growth factor in patients with AMD. This type of growth factor reduces proliferation of blood vessels, thus preventing AMD. Twelve RCTs were included (5,496 participants), comparing pegaptanib, ranibizumab or bevacizumab versus no administration of anti-vascular endothelial growth factor; and a comparison of ranibizumab versus bevacizumab.

#### Ranibizumab versus bevacizumab

- Visual acuity (assessed from the proportion of patients with a gain of 15 letters or more): no difference between the interventions after one year (RR 0.90; 95% CI 0.73 to 1.11; six RCTs; 2446 participants; high certainty of evidence);
- Progression to loss of vision (loss of 15 letters or more): no difference between the interventions after one year (RR 1.00; 95% CI 0.98 to 1.02; six RCTs; 2446 participants; high certainty of evidence);
- Number of serious systemic adverse events: higher with bevacizumab after one year (RR 1.27; 95% CI 1.06 to 1.52; 2597 participants; four RCTs; moderate certainty of evidence).

#### Pegaptanib versus sham

- Visual acuity (assessed from the proportion of patients with a gain of 15 letters or more): favored pegaptanib after one year (RR 2.83; 95% CI 1.23 to 6.52; one RCT; 1,186 participants; high certainty of evidence);
- Progression to loss of vision (loss of 15 letters or more): favored pegaptanib after one year (RR 1.24; 95% CI 1.11 to 1.39; one RCT; 1,186 participants; high certainty of evidence);
- Proportion of participants with serious adverse events: no difference between the interventions after one year, although the estimate was very imprecise because of the low number of events (RR 1.25; 95% CI 0.93 to 1.70; one RCT; 1,190 participants; moderate certainty of evidence).

#### Ranibizumab versus sham

- Progression to loss of vision (loss of 15 letters or more): favored ranibizumab after one year (RR 1.53; 95% CI 1.41 to 1.64; three RCTs; 1,322 participants; high certainty of evidence);
- Proportion of participants with serious adverse events: no difference between the interventions after one year, but the evidence was imprecise and no important differences could be excluded (range of risk ratios [rRR] 0.17; 95% CI 0.01 to 4.24 for ischemic cardiomyopathy; 2.08; 95% CI 0.23 to 18.45 for myocardial infarction; two RCTs; 603 participants; moderate certainty of evidence).

#### Bevacizumab versus standard therapy

- Visual acuity (assessed from the proportion of patients with a gain of 15 letters or more): favored bevacizumab after one year (RR 7.80; 95% CI 2.44 to 24.98; two RCTs; 159 participants; moderate certainty of evidence);
- Progression to loss of vision (loss of 15 letters or more): favored bevacizumab after one year (RR 1.28; 95% CI 1.09 to 1.50; two RCTs; 159 participants; moderate certainty of evidence);
- Proportion of patients with serious systemic adverse events: no difference between the interventions after one year, but this result was very imprecise (RR 2.03; 95% CI 0.19 to 21.85; one RCT; 131 participants; low certainty of evidence).

The review authors concluded that the results indicated that there were benefits from use of anti-vascular endothelial growth factor, for patients with AMD. The assessment of adverse events was impaired by the low number of events.

For further details and to access all the analyses, see the original abstract, available from: https://www.cochranelibrary.com/cdsr/doi/10.1002/14651858.CD005139.pub3/full.

#### 11. Bevacizumab versus ranibizumab

This review^15^ compared the systemic safety of bevacizumab versus ranibizumab and included nine RCTs (3,665 participants). There was no difference in the risk of death between the two drugs (RR 1.1; 95% CI 0.78 to 1.57; eight RCTs; 3,338 participants; moderate quality of evidence). Regarding the number of serious systemic adverse events, no difference was found between the groups (RR 1.08; 95% CI 0.90 to 1.31; nine RCTs; 3,665 participants; low quality of evidence). These results were substantially different from the previous review, which found that use of bevacizumab led to a higher number of serious adverse events. This difference was mainly due to the difference in the number of RCTs included in the analysis (while the previous review only included four RCTs, this review included nine).

The authors of this SR concluded that there were no significant results that could support use of bevacizumab or ranibizumab.

For further details and to access all the analyses, see the original abstract, available from: https://www.cochranelibrary.com/cdsr/doi/10.1002/14651858.CD011230.pub2/full.

### Systemic medications

#### 12. Complement inhibitors

This review^16^ aimed to evaluate complement inhibitors for treating AMD. The authors found only two ongoing RCTs with no results available at time and therefore no numerical data assessing the effects of this intervention were included. So far, there is insufficient data for any conclusion to be reached regarding complement inhibitors for treating AMD and a future update of this review is warranted.

For further details and to access all the analyses, see the original abstract, available from: https://www.cochranelibrary.com/cdsr/doi/10.1002/14651858.CD009300.pub2/full.

#### 13. Interferon alpha

Interferon alpha is an antiangiogenic drug that inhibits migration and proliferation of vascular endothelial cells. This review^17^ assessed the use of interferon alpha for treating AMD and included one RCT (481 participants). In comparison with placebo, use of interferon alpha was associated with worse results, consisting of loss of three or more lines of vision at 52 weeks (OR 1.60; 95% CI 1.01 to 2.53; one RCT; 391 participants). This review was published in 2006 and did not assessed the certainty of evidence.

Further RCTs are needed in order to increase confidence in this estimate. The next update of this review will probably assess the certainty of the evidence using the GRADE approach.

For further details and to access all the analyses, see the original abstract, available from: https://www.cochranelibrary.com/cdsr/doi/10.1002/14651858.CD005138.pub2/full.

#### 14. Statins

Recent studies have shown that AMD and atherosclerosis present some risk factors in common, and that statins may present benefits for patients with AMD. This review^18^ assessed the effects of statins for treating AMD and included two RCTs (144 participants) comparing simvastatin versus placebo among older people who were at high risk of developing AMD (drusen were observed in examinations). Overall, data regarding effectiveness and safety were underreported and the results from the RCTs were not pooled. The main findings were the following:

- Visual acuity: there was no difference between the groups at three months of treatment (decimal visual acuity 0.21 ± 0.56 for simvastatin versus 0.19 ± 0.40 for placebo; 30 participants); at 45 days after the completion of treatment (decimal visual acuity 0.20 ± 0.50 for simvastatin versus 0.19 ± 0.48 for placebo; 30 participants); or at 12 months (42 participants; numbers not provided).
- Drusen score and visual function results were reported to be similar between the groups at 12 months (42 participants), but no effect estimates or confidence intervals were provided.
- Progression of AMD: there was no difference between the groups at three years (OR 0.51; 95% CI 0.23 to 1.09; low certainty of evidence).
- Adverse events: only one RCT reported adverse outcomes, and it was stated that there were no differences between the groups regarding death, muscle aches or acute hepatitis.

The authors of this SR concluded that the current evidence from RCTs was insufficient to confirm that statins had any benefit with regard to preventing or delaying the onset or progression of AMD.

For further details and to access all the analyses, see the original abstract, available from: https://www.cochranelibrary.com/cdsr/doi/10.1002/14651858.CD006927.pub5/full.

### Phytotherapy/vitamins/supplements

#### 15. Antioxidant vitamins and mineral supplements for prevention

Observational studies have suggested that a diet enriched with antioxidant vitamins (carotenoids and vitamins C and E) or minerals (selenium and zinc) may reduce the risk of development and progression of AMD. This review^19^ assessed the effects of taking antioxidant vitamins and/or mineral supplements with regard to prevention of AMD, and it included five RCTs (76,756 participants) with low risk of bias.

#### Vitamin E versus placebo

- Overall risk of AMD: no difference between the groups (RR 0.97; 95% CI 0.90 to 1.06; four RCTs; 55,614 participants; high certainty of evidence);
- Risk of late AMD: no difference between the groups (RR 1.22; 95% CI 0.89 to 1.67; four RCTs; 55,614 participants; moderate certainty of evidence);
- Adverse events: two RCTs reported similar numbers of adverse events for both groups. A third RCT reported that there was higher risk of hemorrhagic strokes in the vitamin E group (HR 1.74; 95% CI 1.04 to 2.91; low certainty of evidence).

#### Beta‐carotene versus placebo

- Overall risk of AMD: no difference between the groups (RR 1.00; 95% CI 0.88 to 1.14; two RCTs; 22,083 participants; high certainty of evidence);
- Risk of late AMD: no difference between the groups (RR 0.90; 95% CI 0.65 to 1.24; two RCTs; 22,083 participants; moderate certainty of evidence);
- Adverse events: use of beta‐carotene was associated with increased risk of lung cancer among people who smoked (high certainty of evidence).

#### Vitamin C versus placebo

- Overall risk of AMD: no difference between the groups (RR 0.96; 95% CI 0.79 to 1.18; one RCT; 14,236 men; high certainty of evidence);
- Risk of late AMD: no difference between the groups (RR 0.94; 0.61 to 1.46; one RCT; 14,236 men; moderate certainty of evidence).

#### Multivitamin (Centrum Silver) versus placebo

Centrum Silver is composed of zinc (15 mg), vitamin E (45 IU), vitamin C (60 mg), beta‐carotene (5000 IU), vitamin A (20% as beta carotene), folic acid (2.5 mg), vitamin B6 (50 mg) and vitamin B12 (1 mg).

- Overall risk of AMD: slightly higher with multivitamin (RR 1.21; 95% CI 1.02 to 1.43; one RCT; 14,233 men; moderate certainty of evidence);
- Risk of late AMD: no difference between the groups (RR 1.22; 95% CI 0.88 to 1.69; one RCT; 14,233 men; moderate certainty of evidence);
- Adverse events: skin rashes were slightly more frequent in the multivitamin group (HR 1.08; 95% CI 1.01 to 1.15; moderate certainty of evidence).

The authors of this SR concluded that vitamin E, beta‐carotene, vitamin C and the multivitamin (Centrum Silver) did not reduce the risk of developing AMD. There was no evidence regarding other antioxidant supplements, such as lutein and zeaxanthin. Although vitamin supplements are commonly assumed to be safe, they may have harmful effects. Hence, sound evidence of benefit is needed before they can be recommended.

For further details and to access all the analyses, see the original abstract, available from: https://www.cochranelibrary.com/cdsr/doi/10.1002/14651858.CD000253.pub4/full.

#### 16. Antioxidant vitamins and mineral supplements for treatment

This review^20^ assessed the effects of taking antioxidant vitamins and/or mineral supplements on the progression of AMD and included 19 RCTs (76,756 participants) with low or unclear risk of bias.

#### Multivitamins versus placebo/no treatment

- Progression to late AMD: less frequent with multivitamins (OR 0.72; 95% CI 0.58 to 0.90; 2445 participants; three RCTs; moderate certainty of evidence);
- Progression of loss of vision (loss of three or more lines on logMAR chart): lower with multivitamins (OR 0.77; 95% CI 0.62 to 0.96; one RCT; 1791 participants; moderate certainty of evidence);
- Quality of life (change in National Eye Institute Visual Function Questionnaire [NEI‐VFQ] score, in which higher scores are better): higher with multivitamins (mean difference [MD] 12.30; 95% CI 4.24 to 20.36; one RCT; 110 participants; low certainty of evidence);
- Adverse events: no difference between the groups regarding mortality (HR 0.87; 95% CI 0.60 to 1.25), but participants in the antioxidant arms more commonly reported presenting yellow skin (8.3% versus 6.0%; P = 0.008; one RCT; 4203 participants; very low certainty of evidence).

#### Lutein and/or zeaxanthin versus placebo

- Progression to late AMD: no difference between the groups (RR 0.94; 95% CI 0.87 to 1.01; one RCT; 6891 eyes; low certainty of evidence);
- Progression to loss of vision (loss of three or more lines on logMAR chart): no difference between the groups (RR 0.98; 95% CI 0.91 to 1.05; one RCT; 6656 eyes; low certainty of evidence);
- Quality of life: no difference between the groups (MD 1.48; 95% CI -5.53 to 8.49 higher; one RCT; 110 participants; moderate certainty of evidence);
- Adverse events: no difference between the groups regarding mortality (HR 1.06; 95% CI 0.87 to 1.31; one RCT; very low certainty of evidence).

#### Vitamin E versus placebo

- Progression to late AMD: no difference between the groups (RR 1.36; 95% CI 0.31 to 6.05; one RCT; 998 participants; very low certainty of evidence);
- Progression to visual loss (loss of three or more lines on logMAR chart): no difference between the groups (RR 1.04; 95% CI 0.74 to 1.47; one RCT; 1179 participants; low certainty of evidence);
- Adverse events: no serious adverse events were reported. No difference between the groups was found regarding withdrawal due to adverse effects (four versus seven), any adverse events (91 versus 83) or ocular adverse events (105 versus 90) (very low certainty of evidence).

#### Zinc versus placebo

- Progression to late AMD: slightly lower with zinc (OR 0.83; 95% CI 0.70 to 0.98; three RCTs; 3790 participants; low certainty of evidence);
- Progression to loss of vision (loss of three or more lines on logMAR chart): no difference between the groups (OR 0.87; 95% CI 0.75 to 1.00; two RCTs; 3791 participants; moderate certainty of evidence);
- Adverse events: gastrointestinal symptoms was more frequently reported as a reason for withdrawal in the zinc group (5/146 versus 2/140; p-value not provided). Anemia was more common in the zinc group (13.2% versus 10.2%; P = 0.004). However, serum hematocrit levels were similar between the groups.

The authors concluded that use of multivitamins, antioxidant vitamins and mineral supplementation may delay the progression of AMD. This finding was based on a single large trial, including only American individuals, and the external validity considering different populations is uncertain. Although vitamin supplements are commonly assumed to be safe, they may have harmful effects.

For further details and to access all the analyses, see the original abstract, available from: https://www.cochranelibrary.com/cdsr/doi/10.1002/14651858.CD000254.pub4/full.

#### 17. Gingko biloba

Ginkgo biloba extracts are used for treating some health conditions, including peripheral vascular diseases, and may present benefits for treating AMD. This review^21^ assessed ginkgo biloba extract for patients with AMD and included two RCTs (119 participants). In these RCTs, it was reported that ginkgo biloba provided some benefits, but there was insufficient data to pool the results.

The outcomes reported in the RCTs were generally different from those of relevance for the review, and the safety results were very sparse. The certainty of evidence was not assessed. Further RCTs are needed in order to reduce the uncertainty of the evidence and to provide a basis for practical recommendations.

For further details and to access all the analyses, see the original abstract, available from: https://www.cochranelibrary.com/cdsr/doi/10.1002/14651858.CD001775.pub2.

#### 18. Omega-3 fatty acids

This review^22^ assessed supplementation using omega-3 fatty acids and included two placebo-controlled RCTs (2343 participants). The main findings were the following:

- Progression to advanced AMD: no difference between the groups (HR 0.96; 95% CI 0.84 to 1.1; two RCTs; 2343 participants; high certainty of evidence);
- Progression to loss of vision (loss of three or more lines): no difference between the groups at 24 months (RR 1.14; 95% CI 0.53 to 2.45; one RCT; 236 participants; moderate certainty of evidence) or at 36 months (RR 1.25; 95% CI 0.69 to 2.26; one RCT; 230 participants; moderate certainty of evidence);
- Adverse events: no difference between the groups (RR 1.01; 95% CI 0.94 to 1.09; two RCTs; 2343 participants; high certainty of evidence).

The authors concluded that there was no evidence of benefits from use of omega-3 among patients with AMD.

For further details and to access all the analyses, see the original abstract, available from: https://www.cochranelibrary.com/cdsr/doi/10.1002/14651858.CD010015.pub3/full.

## DISCUSSION

This review included 18 Cochrane systematic reviews (SRs) that evaluated three surgical techniques, three interventions based on laser/photo/radiotherapy, four different drugs for use in intravitreal injections, three systemic drugs and four complementary interventions for preventing or treating age-related macular degeneration (AMD). The following interventions may present some benefits for AMD: (a) use of bevacizumab, ranibizumab or pegaptanib; (b) laser photocoagulation; (c) photodynamic therapy; and (d) use of multivitamin compounds.

The Cochrane SRs found insufficient evidence to support use of the following: (a) macular translocation (which was also associated with considerable harm); (b) submacular surgery; (c) steroid implantation; (d) radiotherapy; (e) aflibercept; (f) interferon alpha; (g) statins; (h) multivitamins, antioxidant vitamins and mineral supplementation as preventive interventions; and (i) omega-3 fatty acids.

No published RCT was found assessing: (a) use of an implantable ophthalmic mini-telescope device for improving visual acuity after impairment of vision due to AMD (results from an ongoing trial are expected to be published in 2020); and (b) use of complement inhibitors.

Among the 18 SRs included, four did not assess the certainty of the body of evidence based on the GRADE approach, since they were developed before this approach became recommended as mandatory in the Cochrane Handbook. It is strongly desirable that SRs should be updated after two years have elapsed, or more frequently if new studies are available. Indeed, the lack of an approach of this nature for supporting SR conclusions is a factor that limits practical applicability.

We observed an issue involving the comparison between ranibizumab and bevacizumab, which was addressed through two different SRs and led to an overlapping of safety assessments. The first SR focused on the overall effects (benefits and harm) of any intravitreal anti-VEGF drug.^14^ The second SR focused on safety outcomes for the single comparison of ranibizumab versus bevacizumab.^15^ Mainly because of differences between the methodological assumptions used for each SR, the findings regarding serious adverse events were inconsistent between these two reviews. Overlapping of PICOs in Cochrane SRs needs to be avoided, and it is uncommon. Specifically, in this context, considering the debate around off-label use of bevacizumab for treating AMD, a second Cochrane SR was developed in an attempt to address safety concerns.

Additional ongoing Cochrane SRs addressing other interventions for treating AMD will be available over the coming months and may contribute towards expanding the body of evidence available for management of AMD.

Further well-designed and well-conducted randomized controlled trials are still necessary, in order to reduce the uncertainties regarding the clinical questions that surround AMD.

## CONCLUSION

This review found 18 Cochrane systematic reviews that evaluated interventions for preventing or treating AMD. Overall, use of bevacizumab, ranibizumab, pegaptanib, laser photocoagulation, photodynamic therapy and multivitamin compounds may present some benefits for treating AMD. Further randomized controlled trials are still necessary, in order to reduce the uncertainties regarding most clinical questions that surround AMD.
